# Scientific

**Published:** 1889-04

**Authors:** 


### SCIENTIFIC ITEMS.

Since the glacier studies of Tyndall and Agassiz, nothing more interesting in that line has been accomplished than the study of the Muir glacier, by Professor Wright. This is in Alaska, between 58 degs. and 60 degs. The peaks rise 15,000 to 20,000 feet high, and there are many glaciers. But the Muir is the most immense, being two and one-half miles wide, with a front wall of nearly 400 feet. Icebergs eternally snap and crash off this face wall, like the roar of artillery. There is nothing grander in the United States, if, indeed, in the world. The ice moves at the rate of about 70 feet per day. A few miles away the ice deposited during the glacier age is washing away, exposing to the air cedars buried tens of thousands of years ago. Here is a spot for the tourist who desires to see the world in the making of it.—*Ex.*

Californian big trees will have to take a second place as botanical giants now that the ocean has undertaken to beat the land in the size of its products. Capt. John Stone, of the ship "Clever," picked up a sea-weed on the Atlantic near the equator that was 1,500 feet long. It was an alga, and has been identified as a specimen of macroceptis pyrifera.—*Philadelphia Times.*

"Securite" is the name given to a new explosive which is said to be flameless when exploded, and will, it is expected, be of especial value as a substitute for ordinary blasting powder and other explosives in fiery coal mines.

Experiments on the speed of the electric current prove that if a proper conductor could be wound around the globe, a signal parting from any point of it would return to the starting point in one-half a second.

If all the land was leveled off into the sea in the effort to fill up the hollows in the ocean beds, water two miles deep would cover the entire surface of the globe.

**The Canals of Mars.**—From forty careful drawings of this planet at the Lick Observatory in July and August, 1888, showing the details of the canals as seen through the great telescope, none has been seen *doubled*, as asserted and drawn by European observers of late years. The submerged continent had also reappeared in the great telescope in its former contour. Can it be possible that double sight or telescopic ghosts have been troubling the astronomers over the water?

**Sun spots.**—There were only two sun spots during November and December last, this being the year of sun spot minimum. What relation may this have to the unusually mild December and January? —*Scientific Amer.*

**Easy Experiment in Chemistry.**—The *Practical Teacher* gives the following simple experiment in chemistry, which any child can try:

Cut three leaves of red cabbage into small pieces, and, after placing them in a basin, pour a pint of boiling water over them, letting them stand an hour; then pour off the liquid into a decanter. It will be of a fine blue color. Then take four wineglasses—into one put six drops of strong vinegar; into another, six drops of solution of soda; into a third, the same quantity of a strong solution of alum; and let the fourth glass remain empty. Fill up the glasses from the decanter, and the liquid poured into the glass containing the acid will quickly change to a beautiful red; that poured with the soda will be a fine green; that poured in with the alum will turn to a pretty purple; while that poured into the empty glass will remain unchanged.—*Ib.*

**Saccharine Prohibited in France.**—The following is an abstract of the preamble of the bill now before the French chamber prohibiting the importation of saccharine into France: "The attention of the administration has been directed to a new coal tar product known as saccharine. This substance, which differs essentially in its elementary composition from vegetal sugars, possesses much greater sweetening power, a quality that was sure to lead to its being used as a substitute for sugar in many cases. We learned from our consular agencies abroad that factories were being established in certain countries for the purpose of bringing saccharine into competition with beet and cane sugar, not only in France, but also in other neighboring markets. The high cost of that substance seemed to constitute an insuperable obstacle to its general adoption, but lately the situation has changed. It can now be more cheaply produced, and already it is extensively used, mixed with glucose, in the preparation of jams, sirups, and liqueurs. It has, therefore, become an urgent necessity to provide a remedy for the evil, in the interests of the customs receipts and that of the health of the consumer; for it has been shown by the report of Drs. Brouardel, Pouchet, and Ogier, in the name of the consulting committee of hygiene, that saccharine, and the various preparations derived from it, are noxious to health, and ought to be prohibited. Wherefore the government has deemed it expedient to prohibit the importation of saccharine and saccharined substances."—*Ib.*

